# Factors associated with social participation amongst elders in rural Sri Lanka: a cross-sectional mixed methods analysis

**DOI:** 10.1186/s12889-018-5482-x

**Published:** 2018-05-16

**Authors:** Celeste Marsh, Paul A. Agius, Gamini Jayakody, Roshan Shajehan, Chandima Abeywickrema, Kelly Durrant, Stanley Luchters, Wendy Holmes

**Affiliations:** 1 Burnet Institute, Melbourne, Australia; 2 Department of Epidemiology and Preventive Medicine, Monash University, Melbourne, Australia; 3 Judith Lumley Centre, La Trobe University, Melbourne, Australia; 4 International Centre for Reproductive Health, Department of Obstetrics and Gynecology, Ghent University, Ghent, Belgium; 5 Central Province Health Department, Kandy, Sri Lanka; 6 PALM Foundation, Nuwara Eliya, Sri Lanka

**Keywords:** Social participation, Organised activities, Older adults, Low and middle income countries, Healthy ageing, Active ageing

## Abstract

**Background:**

Populations of low and middle-income countries are ageing rapidly; there is a need for policies that support an increase in the duration of old age lived in good health. There is growing evidence that social participation protects against morbidity and mortality, but few studies explore patterns of social participation. Analysis of baseline quantitative and qualitative data from a trial of the impact of Elders’ Clubs on health and well-being in the hill country of Sri Lanka provided an opportunity to better understand the extent of, and influences on, social participation among elders.

**Methods:**

We analysed data from 1028 baseline survey respondents and from 12 focus group discussions. Participants were consenting elders, aged over 60 years, living in Tamil tea plantation communities or Sinhala villages in 40 randomly selected local government divisions. We assessed participation in organised social activities using self-reported attendance during the previous year. Multivariable regression analyses were used to explore associations with community and individual factors. The quantitative findings were complemented by thematic analysis of focus group discussion transcripts.

**Results:**

Social participation in these poor, geographically isolated communities was low: 63% reported ‘no’ or ‘very low’ engagement with organised activities. Plantation community elders reported significantly less participation than village elders. Attendance at religious activities was common and valued. Individual factors with significant positive association with social participation in multivariable analyses were being younger, male, Sinhala, married, employed, and satisfied with one’s health. Domestic work and cultural constraints often prevented older women from attending organised activities.

**Conclusions:**

Elders likely to benefit most from greater social contact are those most likely to face barriers, including older women, the oldest old, those living alone and those in poor health. Understanding these barriers can inform strategies to overcome them. This might include opportunities for both informal and formal social contact close to elders’ homes, consulting elders, providing childcare, improving physical access, advocating with elders’ families and religious leaders, and encouraging mutual support and inter-generational activities. Influences on social participation are interrelated and vary with the history, culture and community environment. Further study is required in other low and middle-income country contexts.

**Electronic supplementary material:**

The online version of this article (10.1186/s12889-018-5482-x) contains supplementary material, which is available to authorized users.

## Background

Populations of low and middle-income countries are ageing rapidly as a result of achievements in reducing fertility rates and extending life expectancy [1]. In Sri Lanka, 13.9% of the population are currently over 60 years, and this is projected to increase to 28.6% by 2050 [2]. Older people contribute significantly to the economy and wellbeing of their families and communities, but poor health and disability can hinder this. Traditional family support for elders is affected by smaller family sizes, more women working outside the home, and migration [3]. There is a need for health promoting interventions for elders to lessen the growing demand for health and long-term care services.

There has long been interest in the role of social relationships in promoting health [4], and the evidence base for the significance of social participation as a protective factor in old age has been growing rapidly [5–9]. A meta-analysis of the extent to which social relationships influence mortality risk in high-income countries found a 50% increased likelihood of survival for participants with stronger social relationships, an influence comparable with the ‘lifestyle’ risk factors [7].

Social participation may influence health and wellbeing through a variety of pathways [10]. Social isolation can result in the release of stress hormones that affect blood pressure, blood lipids, and immunity [11–14]. The hormone oxytocin, which reinforces social bonding, is anti-inflammatory and cardio-protective [15]. Social contacts may provide emotional and practical support, access to information, new skills, peer support in managing chronic conditions [16], greater physical activity, a collective voice for advocacy, joint income generating opportunities and easier access to services [10].

‘Social participation’ has been conceptualised and measured from varied perspectives by researchers from different disciplines. Social scientists have widely adopted the term ‘social capital’ viewing an individual’s social network as an asset. Navarro, however, argues that ‘social capital’ is part of the neoliberal economic discourse, displacing analysis of power and politics [17]. Development workers have long studied ways to facilitate ‘community participation’, including citizen engagement and volunteerism [18]. Some researchers include both formal organised activities and informal social contact, such as chatting with a neighbour [19]. Disability researchers are interested in barriers to participation, so often examine participation in family and work [20]. In recent years, scales have been developed and validated for measuring social participation among groups such as the disabled [21], the mentally ill [22] and older people [23]. Several researchers have noted the variation in how social participation is measured [7, 18, 24].

**Fig. 1:**
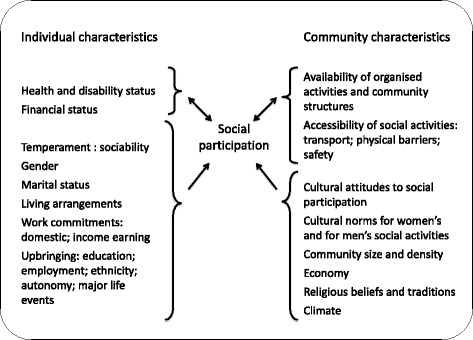
Conceptual model: influences on social participation

It is useful to study the factors that might influence the extent of social participation by elders in different settings. These factors may themselves be influenced by the extent of social participation, and by each other. For example, in some settings younger and fitter elders may have easier access to social activities than ill or disabled elders [6], while in poor communities their need and ability to work may restrict social activities. They can usefully be divided into characteristics of individuals and of their environments [25, 26]. In Fig. 1, we show individual and community characteristics that might influence elders’ social participation, based on the literature.

This study aims to describe the extent and patterns of social participation in organised social activities among elders in a rural Sri Lankan setting, and to explore any associations with individual and community characteristics. We have used the baseline qualitative and quantitative data collected as part of a cluster randomised controlled trial (RCT). The trial, which was part of the ‘Better Vision, Healthy Ageing (BVHA)’ Program (2012–2016), aimed to assess the impact of community level Elder’s Clubs on the health and wellbeing of elders [Trial registration: International Development Impact Evaluations Registry, ID-533c34a8b8734].

## Methods

### Study setting and population

The study was conducted in two of the five administrative divisions of Nuwara Eliya district. This area has a population of 316,213, of whom 35,669 (11.3%) are over 60 years, across 197 Grama Niladhari (local government) Divisions (GNDs) [27]. Tea plantation communities, mostly Tamil, make up 53% of this population; 41%, mostly Sinhala, live in rural villages; and 6% live in urban centres [27]. Nuwara Eliya town is 1889 m above sea level; the climate is cool and damp. Tea plantation residents are a disadvantaged group with poor socio-economic, health and nutrition indicators [28]. Eligible participants were aged 60 years or older and lived in the study area.

### Study design

This was a mixed methods study; we used the baseline questionnaire data (Additional file 1) from the trial as cross-sectional survey data for multivariable analyses. The sample size of 1200 and the number of clusters were determined based on the needs of the cluster RCT. Forty GNDs (clusters) were randomly selected, proportionate to population size. GNDs had a median population of older people of 247 (range: 28–530) living in between one and seven villages or tea plantation communities. A fixed size random sample of 30 eligible participants was then drawn from local government population registers in each GND, with an additional random replacement sample of 10 to allow for migration, death or declined participation. Relevant findings from twelve focus group discussions (FGDs) were used to complement the quantitative results.

### Ethical considerations

For reasons of equity and to avoid selection bias a consenting proxy respondent (family member) answered for elders with impaired cognition, deafness or inability to speak in the quantitative survey. Signed informed consent was obtained; participants unable to write gave their thumbprint, and a witness signed to confirm that the elder understood the study. The Program Elders Advisory Group, made up of elders from the area, was consulted about ethical issues. The study received approval from the Ethics Review Committee of the University of Peradeniya, Sri Lanka, and the Alfred Hospital Research Ethics Committee in Melbourne, Australia (Reference No. 149/13).

### Quantitative component

#### Study procedures

A team of 20 trained data collectors, both Tamil and Sinhala, undertook data collection between August and November 2013. Community leaders invited sampled elders to an appropriate venue; some home visits were also required. After explaining the purpose of the study, the data collectors requested permission to ask the 10 questions of the adapted and translated Abbreviated Mini-Mental State examination (MMSE). Those who scored seven or more were provided with detailed information about the study processes, benefits and risks, and asked to provide informed consent. When participants scored less than seven, a family member was identified as a proxy respondent, and informed consent was requested from both the proxy and the elder. Data collectors of the same sex as the elder administered a comprehensive questionnaire (in Tamil or Sinhala) including questions about their demographic, health, social behaviour, health behaviours, and economic data. Anthropometry and blood pressure were also assessed. The questionnaire had been translated into Sinhala and Tamil and back-translated to check for accuracy and lack of ambiguity. The translated questionnaires had been modified after pre-testing with a group of Tamil elders and a group of Sinhala elders.

#### Data management

Each participant was assigned a unique identification number. The data were double-entered into EpiData version 1.4.2 (EpiData Data Entry, Data Management and basic Statistical Analysis System, Odense Denmark: EpiData Association). Data were compared and validated using the in-built report within the EpiData software. Where appropriate, missing unit record data was compared with matching hardcopy questionnaire responses and corrected accordingly. Initial data cleaning and screening for analysis was undertaken using the Stata statistical software package Version 13.1 (Stata Corp, College Station, TX: StataCorp LP, USA).

#### Study measures

##### Outcome - level of social participation

We asked about frequency of participation in eight organised social activities (Table 2) during the previous year (daily, weekly, monthly, six monthly and once a year or less). We derived an overall score ‘events per year’ (EPY) for each elder, and categorised these as: ‘none or very low’ (attending fewer than three events in a year); ‘low’ (less than monthly attendance); ‘medium’ (monthly to weekly attendance); and ‘high’ (weekly attendance or more). We chose to include only organised social activities in our analyses because influences on organised and informal social activities have been found to differ [19, 29] and organised social activities are more amenable to intervention.

##### Independent factors

The choice of covariates for the analysis was theoretically driven [19, 30, 31].

#### Sociodemographic factors

We asked elders their date of birth and grouped age: 60–69 years; 70–79 years; and 80 years or older. Ethnicity was recorded as Tamil or Sinhala. Elders were categorised as ‘married’ and ‘not married’ (widowed, never married, divorced or separated). Household composition comprised four groups: ‘living alone’; ‘living only with spouse’; ‘living with three or less additional household members (not including spouse only)’; and ‘living with four or more additional household members’. Elders were asked about the number of years at school. We categorised this as ‘no formal schooling’, ‘some primary schooling (1-5 years)’, and ‘some secondary schooling (6-12 years)’. Elders reported whether they were engaged in paid work or not.

#### Disability

We categorised elders as ‘independent in activities of daily living (ADLs)’ if they could eat, dress, wash, use the toilet, take care of their appearance, walk, and get in and out of bed without assistance. Elders were otherwise classified as ‘requiring help with at least one ADL’. The ADL scale was adapted from the Katz Scale [32].

#### Fear of falling

Participants rated their level of concern about falling at social events on a four-point scale [33]. We created a binary variable: ‘not concerned’ (‘not at all’ or ‘somewhat concerned’) and ‘concerned’ (‘fairly’ or ‘very concerned’).

#### Vision

To develop a binary self-reported vision variable, we used responses to the question: “In general, would you say your sight is ‘excellent’, ‘good’, ‘poor’ or ‘blind” and re-categorised this as: ‘poor or blind’ and ‘good or excellent’.

#### Depression

We employed the 7-item Geriatric Depression Scale (GDS-7), which has been validated in an urban Asian setting [34]. We used a cut-off score for depression of three or above.

#### Self-reported health

The question “How satisfied are you with your health” answered on a five point scale from ‘not at all’ to ‘extremely’ is included in the Quality of Life Instrument for the Young Elderly in Sri Lanka (QLI-YES) [35]. Those who responded ‘not at all’, ‘a little’ or ‘somewhat’ were compared with those who responded ‘very much’ or ‘extremely’.

#### Satisfaction with social contact

The QLI-YES includes the question “How satisfied are you with your contact with family and friends?” Those with ‘low satisfaction’ levels (those responding ‘not at all’, ‘a little’ or ‘somewhat’) were compared with those with ‘high satisfaction’ (those responding ‘very much’ or ‘extremely’).

#### Sociability trait

We used a scale developed and validated by Cheek and Buss [36], which measures the sociability trait, or preference to be with other people. This has five positive items scored on a Likert scale from 1(strongly disagree) to 5 (strongly agree). Scores for each item were aggregated to derive a composite measure of sociability.

### Statistical analyses

We used contingency table analyses to provide prevalence estimates of social participation and selected variables modelled in multivariable analyses. In univariable analyses, standard errors and associated 95% confidence intervals around prevalence estimates took account of the GND clustering. Generalized linear latent and mixed modelling (gllamm) [37], was used to estimate multi-level proportional odds logistic regression models exploring correlates of social participation, with a random intercept for sampled GNDs and study participant level covariates estimated as fixed factors. Where the proportional odds assumption was not met for specific factor effects in proposed models (i.e. the independent effects of a factor varied across levels of social participation), gllamm modelling specified covariate specific threshold logit regression models in order to relax the proportional odds constraint. Brant tests [38] and likelihood ratio tests between nested gllamm models (less constrained models relaxing the proportional odds assumption for selected factors) were used to provide statistical inference on whether data met the proportional odds regression assumption. Multivariable proportional odds regression analyses treated GNDs as random factors and therefore modelled the dependency in the sampled divisional clusters directly, providing effect estimates and standard errors for exposures, which took account of GND specific variance in rates of social participation. Random intercept variances from gllamm models were used to estimate both unconditional and conditional (i.e. after estimation controlling for covariates) intraclass correlations (ICC) for level-2 units in order to describe the level of GND specific heterogeneity in social participation. For polytomous exposures, post-estimation Wald tests were undertaken to test for joint effects and statistical differences between exposure groups. A complete case approach was taken and statistical significance assessed at the 5% level.

### Qualitative component

The baseline study included 12 Focus Group Discussions (FGDs) each with 8–10 elders (total 110). We conducted half the FGDs in tea plantation communities, with Tamil retired tea plantation workers, and half in Sinhala rural or peri-urban village communities. We selected participants for each group to be from the same community, speak the same language, and to include a range of ages over 60 years, and both married and widowed elders. All invited elders agreed to take part. The FGDs took place in a temple, dispensary, village hall, or in the home of an elder. Gender may influence discussion dynamics so we conducted four groups with only women, four with only men, and four were mixed. Each group had a facilitator and a note-taker. The trained facilitators were the four Program Officers (community development workers) and two field staff who spoke the same language as the participants and were familiar with the communities. A comprehensive question guide about health and wellbeing was developed based on review of the literature, which included questions about social participation. The local team refined the guide to ensure that the questions were appropriate and relevant. The questions about social participation included the types of social activities elders participated in, benefits perceived and barriers they experience.

The elders gave written informed consent. Facilitators advised the participants that the discussion would be confidential and requested them not to talk about what was discussed with non-participants. The FGDs lasted about one hour. They were audio recorded with permission from the participants.

#### Qualitative data analysis

The recordings were transcribed verbatim and translated into English. The data were organised under themes and sub-themes that emerged from the data, and summarised. The interpretation was checked by the Sri Lankan researchers and facilitators. We have illustrated the findings with verbatim quotes.

## Results

**Fig. 2:**
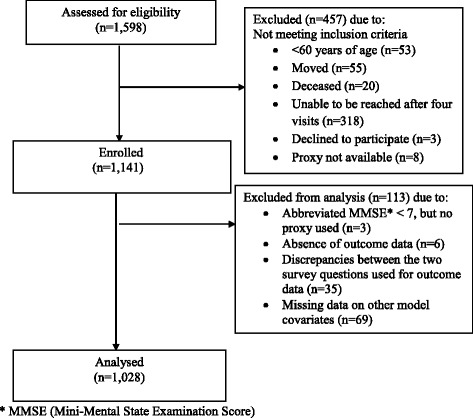
Baseline Data Flow Chart

For baseline interviews, 1141 elders were enrolled (Fig. 2). Figure 2 shows the exclusion pathway from baseline interviews (*n* = 1141) to the final sample for analysis (*n* = 1028). The response rate was 88%. The average number of elders interviewed in each of the randomly selected GNDs was 28.3 (SD = 1.8). For 6% of the sample, a family member answered on behalf of the elder. Of those interviewed, three scored below seven on the cognitive screening assessment using the abbreviated MMSE and did not have a proxy answer questions on their behalf and therefore did not meet the study inclusion criteria. Of the 1132 elders (99.2%) who provided a response on the social participation outcome measure; 35 had discrepancies between the two parts of the social participation question and were excluded from the analysis. The remaining 1028 participants all had complete data for covariates and constitute our population for the analysis. Cases included in the multivariable regression model showed marginally higher levels of social participation to those excluded on the basis of incomplete or unreliable data (OR = 1.58), however, this difference was not statistically significant (Wald χ^2^(1) = 2.02, *p* = 0.16).

### Quantitative sample characteristics

There were 543 women (53%) and 485 men (47%). The average age was 68 years (SD = 6.4; Table 1). The majority of elders (56%) lived in Tamil plantation communities; 33% lived in Sinhala villages; and 11% lived in mixed Tamil and Sinhala communities adjacent to tea plantations. Almost half (49%) the elders lived in households of four or more; only 60 (6%) were living alone. Two thirds of the sample (*n* = 628) were married; 312 (30%) were widowed (11% of the men and 48% of the women), 85 (8%) had never married and three had divorced or separated. About one quarter of the sample had received no formal schooling (*n* = 254); 82% of these were women (*n* = 205). The mean number of years of schooling was 4.1 years (SD = 3.5). Of all elders in the sample, 284 (27.6%) took part in paid work. Just over half of the elders reported low satisfaction with health (54%). Most of the elders (85.9%) were independent in ADLs such as washing and dressing. Over half the sample reported poor vision or blindness (54.5%). The mean score for ‘sociability trait’ was 20.2 (SD = 0.08).

**Table 1:** Descriptive characteristics and social participation among elders, in Nuwara Eliya, Sri Lanka (*n* = 1028). Counts (n) and percent (%)

Factors	n (%)
Community Factors	
Community Type	
Plantation	573 (56)
Village	339 (33)
Mixed communities adjacent to the plantations	116 (11)
Sociodemographic Factors	
Gender	
Female	543 (53)
Male	485 (47)
Age (years)	
60–69	691 (67)
70–79	267 (26)
80 or more	70 (7)
Ethnicity	
Tamil	691 (67)
Sinhalese	337 (33)
Marital status	
Married	628 (61)
Unmarried^a^	400 (39)
Household composition	
Lives alone	60 (6)
Couple living together	133 (13)
Living with 1–3 others (excluding couples)	336 (33)
5 or more members in household	499 (48)
Education (years of Schooling)	
No formal schooling	254 (25)
1–5	479 (47)
6–12	295 (29)
Engagement in paid work	
Yes	284 (28)
No	744 (72)
Activities of Daily Living (ADL)^b^	
Independent	883 (86)
Requiring some help	145 (14)
Concern about falling at social events^c^	
None-little	795 (77)
Somewhat -Very	233 (23)
Vision quality (self-reported)	
Poor vision or blind	560 (54)
Good or excellent vision	468 (46)
Depression^d^	
Yes	361 (35)
No	667 (65)
Self-reported health status^e^	
Low	551 (54)
High	477 (46)
Family connectedness and traits	
Sociability scale^f^ mean total score (SD)	20.2 (0.1)
Satisfaction with contact with family^g^	
Low	261 (25)
High	767 (75)
Social participation (Outcome measure)	
Participation frequency (events per year)	
No/very low participation (< 3)	652 (63)
Low participation (3–11)	132 (13)
Medium participation (12–51)	111 (11)
High participation (52+)	133 (13)

^a^Includes those who reported ‘never married’, ‘separated’, ‘widowed’ or ‘divorced’

^b^Based on the Katz Scale [32]

^c^Based on the question, “How concerned are you about falling when going out to a social event?”

(Falls Efficacy Scale International (FES –I)) [33]

^d^Based on a cut-off score of ≥3 (GDS7) [34]

^e^Derived from responses to the question, “How satisfied are you with your health?”(Quality of Life Index (EQLI)) [35]

^f^Cheek and Bass sociability scale [36]

^g^Derived from responses to question, “How satisfied are you with your contact with family and friends?” (Quality of Life Index (EQLI) [35]

### Patterns of social participation

Participation in organised social activities was generally low, with more than half the sample (*n* = 652, 63%) reporting ‘no’ or ‘very little’ (< 3 EPY) engagement in the past year (Table 1). Of those who reported taking part in at least one organised social activity, the median number of EPY was 13 (IQR = 12–64).

The unconditional intraclass correlation coefficient (ICC) for elders’ social participation indicated that the level of between GND heterogeneity in levels of participation among elders was moderate to high (*ρ*=0.42). The conditional ICC, from multivariable modelling, reduces to an estimated *ρ*=0.10.

Table 2 describes the frequency of participation by activity type and shows religious activities were the most common, with the majority of elders taking part on average once a month. Only 16 elders reported taking part in any sporting activity. Other organised activities mentioned were ‘Funeral society’ (37), ‘Political activities’ (6), and ‘Welfare society’ (1). Thirteen elders reported an unspecified ‘other’ activity.

**Table 2:** Social participation by type of activity (*n* = 804 participation events) for those reporting any participation (*n* = 583 elders): counts (n) and per cent (% elders)

Activity	Participation frequency, n (%)					
	daily	weekly	monthly	6- monthly	≤yearly	*Total*
Community based organisation	0	7(0.7)	58(5.6)	7(0.7)	1(0.1)	73(7.1)
Women’s group	0	10(1.0)	25(2.4)	5(0.5)	2(0.2)	42(4.1)
Youth or children’s group	0	2 (0.2)	8(0.8)	5(0.5)	5(0.5)	20(1.9)
Temple/ church activities	20(2.0)	103(10.0)	176(17.1)	37(3.6)	40(3.9)	376(36.7)
Activities with elders^a^	37(3.6)	46(4.5)	94(9.1)	31(3.0)	12(1.2)	220(21.4)
Sporting activity	0	2(0.2)	4(0.4)	5(0.5)	5(0.5)	16(1.6)
Other^b^	2(0.2)	3(0.3)	37(3.6)	10(1.0)	5(0.5)	57(5.5)

^a^Activities designed specifically for older people

^b^‘Other’ activities reported included ‘funeral society’ (*n* = 37), political activities (6), welfare society (1) and unspecified (13)

In the FGDs, both Sinhala and Tamil elders talked of valuing participation in community affairs: *“It’s important, it will give peace of mind and happiness.”* [Tamil man]. When asked about the types of social activities they participate in, they referred most commonly to religious activities: *“Earlier we got married, we produced and raised our children and didn’t have much time for these religious activities, so this is a good time to follow these religious activities in our life.”* [Sinhala man]; *“In our temple, if anything happens, they call me. I am good at art, therefore I get opportunities to perform and this makes me happy.”* [Tamil man]. Many elders felt they gain respect when they participate in religious rituals: *“We can observe ‘sil’ (religious alms giving) and we can get respect from others, and as older people we are always thinking about religious things like alms-giving and meditation and participate in those activities.”* [Sinhala man]. Several elders talked about their special role at ceremonies such as funerals and weddings: *“As women who are living in this village we attend to puberty ceremonies, funerals, alms-givings and we help in such kind of activities.”* [Sinhala woman].

Helping others was a common theme. Shramadana activities (sharing one’s time and energy for the welfare of all) are common in Sri Lanka and were conducted by both Sinhala and Tamil elders. Alms-giving, visiting the sick and sweeping temples were also often mentioned. The elders also talked about helping their neighbours by providing meals, helping them to take medicines, and consoling them when bereaved. Inter-generational activities were important to several elders as a way of passing on their knowledge and skills: *“I was teaching kavadi folk dance to five students – those are good cultural things – it should not be destroyed – the current generation is more interested in Indian dance. They don’t have our skills now.”* [Tamil man]. Other social activities mentioned included going on excursions, especially to religious sites, gardening, political activities, and getting together with friends.

### Factors associated with taking part in organised activities

In exploring the extent to which the multivariable model was specified correctly (i.e. testing the proportional odds assumption), a Brant test from normal proportional odds regression analyses showed that the assumption of proportional odds for the specified model was not reasonable given the data (χ2(40) = 75.7; *p* = 0.001) and that gender (χ^2^(2) = 24.2, *p* < 0.001) exhibited a non-proportional effect. This was supported statistically by likelihood ratio testing from gllamm modelling, which showed that partial relaxation of effect proportionality (i.e. for gender) showed significantly better fit than the fully constrained model (LR χ^2^(2) = 22.1, *p* < 0.001).

Factors found to have significant positive univariable and multivariable associations with social participation included: living in a village, Sinhala ethnicity, younger age, male gender, being married, employed in paid work, and being satisfied with one’s health (Table 3). Being aged over 80 years was negatively associated with taking part in organised social activities.

**Table 3:** Factors associated with social participation among elders from multi-level proportional odds regression analyses: unadjusted (OR) and adjusted (AOR) odds ratios, 95% confidence intervals (95% CI) and probability values (*p*-values) (n = 1028)

Factors	Proportional Odds				Unconstrained Effects					
	OR (95% CI)	*p*-value	AOR (95% CI)	*p*-value	Low participation		Medium Participation		High Participation	
					AOR (95% CI)	*p*-value	AOR (95% CI)	*p*-value	AOR (95% CI)	*p*-value
Gender (Male)					1.40(0.95,2.06)	0.090	2.17(1.43, 3.29)	< 0.001	4.45(2.71, 7.32)	< 0.001
Community Type										
Plantation	1.00		1.00							
Village	18.9 (12.5, 28.5)	< 0.001	7.5 (3.6,15.5)	< 0.001						
Mixed Community	1.12 (0.68, 1.86)	0.646	1.86 (0.86, 4.06)	0.116						
Age										
60–69 years	1.00		1.00							
70–79 years	0.85 (0.61, 1.18)	0.328	0.78 (0.55, 1.12)	0.182						
80 or more	0.38(0.21, 0.69)	0.001	0.44 (0.22, 0.86)	0.017						
Ethnicity (Sinhala)	5.55 (3.23, 9.57)	< 0.001	3.95 (2.18, 7.18)	< 0.001						
Marital status (married)	2.31 (1.69, 3.16)	< 0.001	1.49 (1.03, 2.16)	0.033						
Household Composition										
Lives alone	1.00		1.00							
Couple living together	1.54 (1.04, 2.29)	0.033	0.85 (0.41, 1.74)	0.653						
1–3 other members^a^	1.06 (0.79, 1.44)	0.688	0.83 (0.43, 1.57)	0.561						
5 or more members	0.80 (0.59, 1.07)	0.132	0.73 (0.38, 1.38)	0.328						
Years of Schooling										
No formal schooling	1.00		1.00							
1–5	0.85 (0.63, 1.14)	0.266	0.97 (0.64, 1.47)	0.891						
6–12	1.86 (1.36, 2.55)	< 0.001	1.02 (0.65, 1.61)	0.930						
Engagement in paid work	2.40 (1.76, 3.28)	< 0.001	1.57 (1.11, 2.21)	0.010						
Requiring help with ADL/s^b^	0.46 (0.31,0.74)	0.001	0.74 (0.46, 1.22)	0.240						
Concern about falling at social events^c^ (quite or very concerned)	0.83 (0.59, 1.16)	0.283	0.91 (0.61,1.35)	0.638						
Vision quality, self-reported (poor or blind)	0.67 (0.50, 0.89)	0.006	0.86 (0.63, 1.17)	0.333						
Depression^d^	0.71(0.52, 0.98)	0.035	0.85 (0.60, 1.20)	0.354						
Health Status (High)^e^	1.63 (1.23, 2.17)	0.001	1.47 (1.07, 2.02)	0.017						
Sociability score^f^	1.06(1.00, 1.13)	0.052	1.03 (0.97, 1.10)	0.354						
Satisfaction with access to family and friends (High)	1.12 (0.80, 1.56)	0.526	1.08 (0.74, 1.56)	0.686						

Model cut-points - k_1_ = 4.1 k_2_ = 5.4, k_3_ = 7.0

^a^Excluding couples living together

^b^Based on the Katz Scale [32]

^c^Based on the question, “How concerned are you about falling when going out to a social event?”(Falls Efficacy Scale International (FES –I)) [33]

^d^Based on a cut-off score of ≥3 (GDS7) [34]

^e^Derived from responses to the question, “How satisfied are you with your health?”(Quality of Life Index (EQLI)) [35]

^f^Cheek and Bass sociability scale [36]

Several other variables including vision, education level, requiring assistance with ADLs, household composition and depression had significant univariate associations with social participation, however, the effect was not significant in the adjusted model (vision: Wald χ^2^(1) = 0.86, *p* = 0.33; assistance with ADLs: Wald χ^2^(1) = 0.74, *p* = 0.24; educational level: Wald χ^2^(2) = 0.07, *p* = 0.96; household composition: Wald χ^2^(3) = 1.29, *p* = 0.73; depression: Wald χ^2^ (1) = 0.71, *p* = 0.35). ‘Satisfaction with social contact’ (Wald χ^2^(1) = 1.08, *p* = 0.69), ‘sociability’ (Wald χ^2^(1) = 1.03, *p* = 0.35) and ‘fear of falling at social events’ (Wald χ^2^(1) = 0.91, *p* = 0.64) were not significantly associated with social participation.

When elders with proxy respondents were excluded from the analyses, two univariate associations dropped below the significance level (*p* = 0.05); assistance with ADLs (OR = 0.64, 95% CI = 0.4–1.04) and age (> 80 years: OR = 0.56, 95%CI = 0.29–1.10). In the adjusted model, the association with age remained non-significant (Wald χ^2^ (2) = 5.67, *p* = 0.06) while the association with being married (Wald χ^2^ (1) = 1.47, *p* = 0.05) and with paid work (Wald χ^2^ (1) = 1.43, *p* = 0.05) were also attenuated with the removal of proxy responses.

#### Community factors

Living in a village as compared to a plantation community had a highly significant effect on participation (OR = 18.9, 95%CI = 12.5–28.5). This association was attenuated when other factors were controlled for (AOR = 7.5, 95%CI = 3.6–15.5). A significant difference in the effect of residing in a village compared to in a mixed Tamil and Sinhala community adjacent to the plantations was also found (Wald χ^2^(1) = 10.43, *p* = 0.001). Sinhala elders (mostly living in villages) were much more likely than Tamil elders (mostly living in plantation communities) to have higher levels of social participation (AOR = 3.95, 95%CI = 2.18–7.18). Further analyses exploring the interaction between community type and ethnicity in terms of participation revealed no differences in the effect of ethnicity across community type (Wald χ^2^(2) = 2.20, *p* = 0.33).

In the FGDs there was some suggestion that traditional villages may be more socially cohesive than tea plantation communities *“We have unity among villagers so most of the time they get together and try to help each other.”* [Sinhala man]. A Tamil man talked of a lack of trust, and others in the group agreed: *“We don’t expect anything from them [neighbours] because they won’t do it – we do not trust them.”* [Tamil man]. We did not hear of any social tensions between different ethnic groups: *“He’s a Muslim, he’s a Christian, he’s a Hindu – we don’t discriminate.”* [Tamil man]. Several elders indicated that lack of organised activities prevented them socialising: *“I have noticed that there are few opportunities in this village that we can share our problems and stresses. I think it causes mental stress.”* [Sinhala elder].

#### Individual factors

##### Gender

Independent of other factors, men had higher levels of participation than women. However, the degree to which this was the case varied by level of participation. There was little difference in men and women’s participation levels when comparing very low levels of participation (AOR = 1.40, 95%CI = 0.95–2.06), while at higher participation levels, the difference between men and women was greater (High participation: AOR = 4.45, 95%CI = 2.71–7.32).

In the FGDs, a strong theme was that gender roles make it more likely that men have social contacts: *“Women are more engaged with domestic work like child care, cooking, sweeping, home gardening, and most men don’t do domestic things, they are more willing to do public things like doing outside jobs, attending meetings, and community activities like shramadana.”* [Sinhala woman]. One Sinhala man, when asked about women socialising, said: *“They have enough time, they can talk with each other. They have more than enough time to talk with each other.”* However, the women replied*: “No, we don’t have enough time because we have to look after our grandchildren in the daytime. Sometimes we have to help with agricultural activities then we can meet our friends there, while working in the paddy fields we can talk with each other and we can enjoy our past experiences.”* [Sinhala woman]. Another Sinhala older woman noted that *“Older women are more shy and more frightened to engage with social actions.”*

However, there were several suggestions from Sinhala elders that gender roles are changing *“Earlier women were doing domestic work and men went outside and worked there. But now, women are engaging with income generation activities and they have got used to going out and men are looking after children in the house.”* [Sinhala woman].

##### Marital status

Being married was positively associated with social participation (AOR = 1.49, 95%CI = 1.03, 2.16). When asked in the FGDs how society reacts towards someone when their spouse dies, one Tamil widow said: *“They just isolate us and will not let us come for good things and events in the village.”*, but several recognised the loneliness of widowhood: *“The most important thing is we should not allow them to feel that they are alone. We have to console them and we have a responsibility to do those things.”* [Sinhala woman].

##### Age

Compared to those aged 60–69 years, elders aged 80 years and older were less likely to participate (AOR = 0.44, 95%CI = 0.22–0.86). Those aged 70–79 years also showed lower levels of participation (AOR = 0.78, 95%CI = 0.55–1.12) but this difference was not statistically significant (*p* = 0.182). There was no difference in participation between elders aged 70–79 and those aged 80 years and older (Wald χ^2^(1) = 2.76, *p* = 0.097).

##### Employment and financial status

Being employed was positively associated with higher levels of participation (AOR = 1.57, 95%CI = 1.11–2.21). While financial status was not assessed in the multivariable analyses, during the FGDs many elders talked about their poverty, which prevents them participating in their communities: *“And older people don’t have enough money. Earlier they had a lot of money, but now they don’t have even a single cent in their hands. Then they think about that and ask support from children and they are very helpless if their children don’t give money to them.”* [Sinhala woman].

##### Health

Being satisfied with one’s health (AOR = 1.47, 95%CI = 1.07–2.02) was positively associated with higher levels of participation. Poor health and disabilities, including vision impairment, were often mentioned by the elders in the FGDs as a cause of social isolation: *“Sometimes we can’t go to meet our friends because now we have got some illnesses.”* [Sinhala woman] and *“In the night we can’t go and meet them because of our vision problems.”* [Tamil man].

##### Inter-generational relationships

Some elders mentioned family conflict and lack of autonomy as factors that restrict their participation: *“In some houses some family members do not allow elders to attend a meeting or go to a temple. They have compelled elders to stay at home. But children go on trips and come back. When elders see that they get upset and it is also a kind of domestic violence.”* [Sinhala woman].*“We have more freedom than in our earlier life, but some elders don’t have freedom like us.”* [Sinhala man]. One older woman noticed a generation gap: *“Sometimes there is a generation gap between youngsters and us. Sometimes their ideas do not match with our ideas. That is a problem sometimes when we do social activities.”* [Sinhala woman].

## Discussion

Designing interventions that aim to increase social participation among older people in low-income settings is a relatively new strategy in international public health. The number of studies of social participation among older people in high-income countries has increased exponentially during the past decade, but we have found few from low or middle-income countries. Local studies such as this would be useful to government officials and civil society in planning interventions to increase social participation.

There were several key findings. First, in general, elders participated very little in organised activities but participation was higher in the rural villages than in the plantation communities. Second, context has a large influence on patterns of social participation. Third, we identified several characteristics of individuals associated with higher levels of participation: male gender, being married, being employed, having good health, and being in the ‘younger’ old age group.

### Community level factors associated with social participation

Clearly, participation in organised social activities depends on the availability of such activities. Tea plantation workers and their families live in poor and crowded conditions. The Tamil elders today are second or third generation descendants of workers brought by the British from India. The migration disrupted their traditional village culture, which was based on family and communal agriculture with many cooperative activities. Plantation residents lacked schools and other community structures and facilities, and were isolated from each other and from nearby towns and villages, without transport. On the other hand, communal life has remained important in the traditional Sinhala rural villages, centred around the temple and religious rituals. These different histories help to explain the difference in availability of organised opportunities for social interaction and the observed strong association of community type with social participation, independent of ethnicity. Other plantation communities in poor countries are likely to have similarly limited opportunities for socialising and may need different approaches to encourage and enable social contact among elders. We have found that when community Elders’ Clubs are established many of the elders in both types of communities are keen to engage, especially in relation to activities that contribute to their communities [10].

A recent meta-analysis of studies in high-income countries identified contextual factors associated with elders’ social participation outside the home including friendliness of neighbours, perceived social support, land-use diversity, street walkability, civil protection and transportation services [25]. These factors are also likely to play a role in low and middle-income countries; an enabling environment is important if elders with disabilities are to participate in their communities. There is a growing movement for age-friendly communities and cities, but most documented experience is from high-income countries [39]. Elders themselves, with young people and local government, can get together to make their community more ‘elder friendly’ [40]. In both cultural groups, religious activities were especially valued by elders and this was also found in a Jamaican study, the authors noting the significant potential for faith-based organisations in promoting active ageing [41].

### Individual characteristics associated with social participation

The consequences of gender inequality and different gender roles help to explain why older men take part in organised activities more than older women in both types of community. Although women have had higher status in Sinhala Buddhist culture than in many South Asian cultures [42], men tend to have greater autonomy than women, and greater access to money for social activities. In the Tamil culture women generally have lower status than men, and this has been reinforced by plantation life and conditions [43]. In the FGDs, both men and women noted women’s heavy burden of domestic work as a reason for not being able to attend social activities. It is also possible that older women’s desire for social contact may be met more through informal activities, chatting with their friends while undertaking domestic tasks such as shopping, caring for grandchildren, and fetching water or firewood.

Studies in Sri Lanka [44], Pakistan [30], Australia [29], the Netherlands [19] and the UK [45], have also found that older women tend to participate less than older men in organised activities, but more than men in informal activities. However, the study in Jamaica found that men were more likely to visit and be visited by friends [41]. Studies investigating the link between social participation and health and wellbeing in older people suggest that men and women may also benefit differently [4, 46].

It is important that gender is taken into account when planning opportunities for social interaction, that women are consulted and encouraged, and that arrangements are made for childcare. Further studies are needed of how to foster older women’s participation and the extent to which informal socialising protects their health and wellbeing, compared to taking part in organised activities.

Widowed elders may be less likely than married elders to take part in social activities because they have more domestic responsibilities, may have less money, be more reluctant to go out to organised activities on their own, or may fear societal disapproval. Tamil widows may be ostracised and their lives viewed as being over [43]. A Pakistan study also found that being widowed was associated with less social participation [30] and married Jamaican elders were more likely to attend organised social activities, attributed to increased access to resources and larger social networks [41].

That the minority of elders who undertake paid work had greater rates of social participation is likely linked to better income, education, health, status, and contacts. Amongst those living with disabilities in Brazil, those employed had higher social participation [31]. This is likely to be context dependent: a German study of older people’s social participation found that full-time employment was negatively associated with both organised and informal social participation [47], but in the Netherlands, no association was found [19].

Studies of older people from Jamaica and high-income countries have found greater social participation among those with more education [19, 41]. Gesthuizen has suggested that schooling socialises children, influences their norms and values, and encourages them to become interested in communal and societal matters [48].

It is difficult to speculate on the reasons why, in this setting, the association between education and social participation was attenuated when other factors were controlled for. Schools were being introduced to the plantation sector when these elders were children, and the quality of education was comparatively poor. The younger elders were more likely than the older ones to have attended school, and for more years. The generally lower education level amongst these plantation elders compared to those in high-income countries could play a role in the observed findings.

Other researchers have also found that ‘the oldest old’ are less likely to participate in organised social activities [30, 44]. Possible explanations include poverty, transport difficulties, a need to be accompanied, ageism, withdrawal from social life, and lack of autonomy. However, among older people in Jamaica, Willie-Tyndale et al. did not find an association with age [41]. This may be because their measurement of social participation included ‘visiting friends regularly and being regularly visited by friends’ as well as ‘meetings of organised social groups’. It might be that the oldest old are more likely to have informal social contacts than to attend organised activities.

The relationship between health status and participation is complex. Social participation contributes to better outcomes for health and well-being, but poor physical or mental health may restrict participation [19, 41, 49]. Health status is also on the causal pathway for other variables that may influence social participation, such as poverty, depression, widowhood [50], and vision impairment. We investigated self-reported satisfaction with health (a predictor of morbidity and mortality [51, 52]), independence in ADLs, depression and self-reported vision. These were all strongly associated with social participation, but the association was reduced after adjustment for other variables. This might in part be because health and disability act as intermediate variables. The reporting of multiple chronic conditions was negatively associated with frequency of social participation among Pakistani elders [30].

The positive associations for paid work and for marital status were attenuated when data from proxy respondents were excluded. This likely reflects the relatively greater importance of these factors in terms of participation for elders in this group, who were more likely to be older and need help with ADLs than those who answered for themselves.

Studies of older people with impaired vision from high-income countries have also found that reduced vision restricts social interactions [53–56]. However, it is difficult to compare the impact of vision loss in richer countries, where treatment is more available and few lack access to glasses, with that in low-income countries [57]. That the negative association of poor vision with social participation was not significant when adjusted for other factors may be the result of over-adjustment [58]. Prevalence of vision impairment increases with age and is higher among women [59]. The prevalence of vision impairment in these communities is high, and there are many barriers to access to affordable treatment. Those with poor vision may be less physically active, less able to attend for health care, and less able to manage chronic conditions. Increased social participation could be a benefit of improving equitable access to low-cost, cost-effective cataract surgery and spectacles [57]. For those whose vision loss cannot be treated, low vision services are also needed to facilitate their social contact and participation [60, 61].

Poor health and disability are significant risk factors for depression; conversely depression can result in illness and disability [62–64]. We did not find significant association between social participation and depression. There have been many studies of the links between mental health and social ties in older age in high-income countries [65, 66]. Most find that social support and social participation are protective, especially for older women [46], although it is difficult to identify the direction of causality [65]. Some studies found that social participation may even increase the risk of depression for older women, with the suggestion that a larger social network increases opportunities for stressful as well as supportive relationships [66]. It is possible that our finding reflects effects in both directions.

Several of the factors that influence social participation in old age are likely to change for different generations of elders, for example, as a result of changes to mortality, fertility, migration, and the economy [19]. It is important to consider cohort effects when planning social interventions for elders, and to monitor rates of social participation at intervals. Smaller families, migration and greater female participation in the workforce reduce opportunities for social contact for elders; community organisations could play an important role in addressing this [10, 41, 67].

### Strengths and limitations

Strengths of this study included the use of mixed methods, the large, community-based probability sample, and administration of the study by the local BVHA program team.

This analysis also has several limitations. While very few of the elders declined to participate, some on the sample list could not be found, and the additional random sample list was then used. Compared to 2011 census data for the district, our sample was somewhat younger, with half as many in the 80+ age group and more in the ‘young-old’ age group. This may be because the oldest age group often needed a home visit by the data collectors, and some may have been missed. It may also be that census workers missed some of the working young-old group. This means that our estimates for rates of social participation may be high. Although the questionnaire used validated scales, we cannot be sure how Sri Lankan elders from a poor and isolated area understood the questions. Six per cent of the elders required a family member to answer on their behalf; we cannot be sure of the validity of their answers. We asked about frequency of participation in organised activities, but not the extent or quality of social relationships. Comparison with other studies is complicated by different definitions of ‘social participation’, and by varied patterns of social support in different cultural contexts.

## Conclusions

The evidence that social participation contributes to health and wellbeing through a range of pathways is strong. Understanding the factors that are associated with social participation is important to ensure a greater proportion of populations can benefit. The WHO World Report on Ageing and Health emphasises that public health policy must serve to break down the many barriers to the social participation of older people and provides many relevant recommendations [1]. Older people themselves can contribute to overcoming the barriers to participation experienced by themselves and their peers.

We found that those most likely to benefit from greater social contact are those most likely to face barriers: older women, the oldest old, the poorest and those in poor health. Our findings emphasise that the factors associated with social participation are interrelated and vary in different contexts. They show the importance of gathering information locally when planning to enable and encourage older people to participate socially.

## Additional file

Additional file 1: English version of the Baseline Questionnaire used for the quantitative component of the study. (PDF 443 kb)

## Abbreviations

ADL: Activity of Daily Living
AOR: Adjusted Odds Ratio
BVHA: Better Vision and Healthy Ageing
EPY: Events per Year
FGD: Focus Group Discussion
GDS-7: 7-item Geriatric Depression Scale
gllamm: Generalised linear latent and mixed modelling
GND: Grama Niladhari (local government) Divisions
ICC: Intraclass correlations
IQR: Interquartile Range
MMSE: Mini-Mental State examination
OR: Odds Ratio
QLI-YES: Quality of Life Instrument for the Young Elderly in Sri Lanka
