# Supplementary material for: Factors associated with social participation amongst elders in rural Sri Lanka: a cross-sectional mixed methods analysis
Source: BMC Public Health. 2018 May 16;18:636. doi: 10.1186/s12889-018-5482-x (PMC5956789; doi:10.1186/s12889-018-5482-x)
Supplement: Supplementary file 1 — English version of the Baseline Questionnaire used for the quantitative component of the study. (PDF 443 kb) [file 12889_2018_5482_MOESM1_ESM.pdf]

Burnet Institute and Fred Hollows Foundation
Better Vision, Healthy Ageing

BASELINE SURVEY QUESTIONNAIRE

AA1. *Study Identification Number:* ☐☐☐☐☐

AA2. *Estate division:*……..……………………….or *Village:*……..…………………………………….

AA3. *Date of interview:* ______/______/________

AA4. *Abbreviated Mini Mental State Score:* ☐☐

Question number	***Score***	***Question number***	***Score***
1.		6.	
2.		7.	
3.		8.	
4.		9.	
5.		10.	

Notes to interviewers are in italics. Anything in normal print should be read out to the person being interviewed. Anything in italics should not be read out.

Participant Information Sheet read out and Consent Form signed: ☐

AA5. *Who is answering the questions?*
Elder participant 1☐ ▶ Skip to AA8
Family member (proxy respondent) 2☐

AA6. *If family member answering on behalf of the elder, what is their relationship to the elder? Do* ***not*** *record their name here:*

____

AA7. *What is the sex of the family member?:*
Male 1☐
Female 2☐

AA8. *Is anyone else present during the interview?* *Yes / No*

If yes, what is their relationship to the elder?

____

Section A. Personal details

I would like to begin by asking you some questions about yourself.

A1. Sex:

Female	1	☐
Male	2	☐

A2. What is your age (last birthday)? ☐☐

A3. Date of birth (if known): ☐☐ ☐☐ ☐☐☐☐

Day Month Year

Not known: 98 ☐

A4. What is your marital status?

Never married	1	☐
Married	2	☐
Widowed	3	☐
Divorced	4	☐
Separated	5	☐

A5. Have you retired from work?

Read all responses

Yes	1	☐
No, work full-time	2	☐ ▶
No, work part-time	3	☐ ▶

If no, skip to AA7

A6. If retired, at what age did you retire? ☐☐

Don't know 89 ☐

A7. What has been your **main** occupation in life?

Tick one only

Tea picker	1	☐
Factory worker	2	☐
Office staff	3	☐
Sundry worker	4	☐
Rice farmer	5	☐
Vegetable farmer	6	☐
Casual labourer	8	☐
Other	9	☐

……………………………………..

SID: Date:

Section B. Details of household and family

Now we would like to ask about who you live with, and about your family.

B1. Do you live alone?

Yes 1☐ ► Skip to B5

No 2☐

B2. How many people are there in your household, **not** counting yourself? ☐☐

B3. *Do not ask if widowed* – Do you currently live with your husband / wife?

Yes 1☐

No 2☐

B4. What is the age group and sex of each person in your household? Can we please start with the youngest? Do not include yourself.

Tick the boxes in the table below with the information the elder gives to you about the members of their household. Do not write down their names.

Household member	Age group						Sex	
	0-4	5-14	15-24	25-49	50-59	60+	Male	Female
1								
2								
3								
4								
5								
6								
7								
8								
9								
10								
11								

B5. Has your spouse or any close family member died in the past 2 years?

Yes 1☐

No 2☐ ► *If no, skip to B7*

B6. I am sorry to hear that. Could you tell me who it was that died?

Write down the relationship here, e.g. spouse; sister; son; brother.
If necessary, there is space for more than one death.

1. …………………………………….
2. …………………………………….
3. …………………………………….

B7. Do you live….

Read all responses

in a room in a line?	1☐
in a mud-brick house?	2☐
in a wooden house?	3☐
in a brick, stone or cement block house?	4☐
Other…..…………………………………	9☐

B8. How many sons do you have (not including sons-in-law)? ☐☐

B9. How many daughters do you have (not including daughters-in-law)? ☐☐

B10. How many of your sons and/or daughters have moved away? ☐☐ ▶ If none, skip to C1

B11. Where did they move to?

To another estate	☐☐
To Colombo or somewhere else in Sri Lanka	☐☐
Overseas	☐☐
Don't know	89☐

SID: Date:

Section C. Education

Next we would like to ask about your schooling.

C1. Have you ever attended school? Yes 1☐ No 2☐ ► If no, skip to C3

C2. If yes, how many years did you have at school in total? 1☐☐

C3. I am going to show you a sentence written on a card. Can you tell me what is written on this card? *Show the elder the card with the sentence and ask them to read the sentence aloud.*

- Able to read whole sentence 1☐
- Able to read only parts of sentence 2☐
- Cannot read at all 3☐
- Vision too poor to tell 4☐

C4. What languages can you speak well enough to have a conversation?

- Tamil only 1☐
- Sinhala only 2☐
- Tamil + Sinhala 3☐

.

Section D. Health status

The next questions are about your health and well-being.

D1. Can you please tell me if you are currently suffering from any of the following chronic symptoms ('chronic' means you have had the symptom for one month or longer)
Show the elder the showcard for question D1 and ask "Please tell me for each symptom, whether it is no problem, mild, moderate or severe" - *and circle the appropriate number.*

Symptom	No problem	Mild	Moderate	Severe*
A. Joint pains (arthritis)	1	2	3	4
B. Backache	1	2	3	4
C. Difficulty with hearing	1	2	3	4
D. Difficulty with sight	1	2	3	4
E. Shortness of breath	1	2	3	4
F. Chronic cough	1	2	3	4
G. Chronic cough with blood*	1	2	3	4
H. Wheezing	1	2	3	4
1. Chest pain	1	2	3	4
J. Constipation	1	2	3	4
K. Tiredness	1	2	3	4
L. Skin rash	1	2	3	4
M. Toothache	1	2	3	4
N. Weakness or paralysis from stroke	1	2	3	4
O. Epileptic fits (at least one fit in past 6 mths)	1	2	3	4
P. Falls (more than one fall in the past mth)	1	2	3	4
Q. Leaking of urine	1	2	3	4
R. Loss of weight	1	2	3	4
S. Dizziness	1	2	3	4
T. Any other symptoms or pains?<br>List:	1	2	3	4

**NB. If the elder reports any of these symptoms as severe, or is coughing blood, and has not already seen and been treated for the problem, explain that, because this might be serious, you would like to help them to see a doctor. Complete two copies of the referral form, and give one to the participant or carer, and keep the other one securely in your bag.*

SID: Date:

D2. Has a doctor ever told you that you have:

A) Diabetes?	Yes	1☐
	No	2☐
	Don't know	89☐
B) Heart disease?	Yes	1☐
	No	2☐
	Don't know	89☐
C) High blood pressure?	Yes	1☐
	No	2☐
	Don't know	89☐
D) Stroke?	Yes	1☐
	No	2☐
	Don't know	89☐
E) Cancer?	Yes	1☐
	No	2☐
	Don't know	89☐
E) Asthma (chronic obstructive airways disease)?	Yes	1☐
	No	2☐
	Don't know	89☐

D3. Have you had any admissions to hospital in the past 6 months?

If yes, complete the table below

Yes 1☐

No 2☐

Don't know 89☐

Type of illness or operation	Name of hospital	Number of nights

Be aware that the following questions (D4 – D8) are sensitive. Read out the possible responses, and then read them again, slowly

D4. A. How often do you experience urine leakage? *Ask each response in turn*

Never 0☐

Less than once a month 1☐

A few times a month 2☐

A few times a week 3☐

Every day and/or night 4☐

B. How much urine do you lose each time? *Ask each response it turn*

None 0☐

Drops 1☐

Small splashes 2☐

More 3☐

D5. How often do you experience leakage of stools? *Ask each response in turn*

Never 0☐

Rarely (< once in past 4 weeks) 1☐

Sometimes (< once a week, but once or more in the past 4 weeks) 2☐

Often or usually (< once a day but once a week or more) 3☐

Always (once or more per day) 4☐

SID: Date:

D6. How much does leaking of urine or stools affect your quality of life?

Read all responses

Not at all	1	☐
A little	2	☐
Somewhat	3	☐
A great deal	4	☐

D7. How much does leaking of urine or stools cause you to limit your social activities?

Read all responses

Not at all	1	☐
A little	2	☐
Somewhat	3	☐
A great deal	4	☐

D8. In general would you say that your sight is?...

Read all responses

Excellent	1	☐
Good	2	☐
Poor	3	☐
Blind	4	☐

NB. 'Blind' means "No more sight than the ability to tell light from dark, and the general direction of a source of light". If the elder says 'blind' please check if they are able to see more than just light and dark. If they can see better than this tick 'Poor'.

D9. When was the last time you had your eyes examined by a professional?

Never	0	☐
Less than one year ago	1	☐
1 – 2 years ago	2	☐
2 – 5 years ago	3	☐
More than 5 years ago	4	☐
Don't know	89	☐

D10. Do you currently own a pair of spectacles that you use to help you to see?

Yes	1	☐
No	2	☐

D11. *Ask this question to all elders. For those who use spectacles, ask them to answer how difficult performing these tasks are when they are wearing their spectacles.*

Because of your vision, how much problem do you have in:						
	No problem at all	Slight problem	Some problem	Serious problem	Cannot do this because of your vision	Not applicable
1. Climbing stairs?	1	2	3	4	5	0
2. Making out the bumps and holes in the road when walking?	1	2	3	4	5	0
3. Seeing if there are animals or vehicles when walking?	1	2	3	4	5	0
4. Finding your way in new places?	1	2	3	4	5	0
5. Finding your way indoors?	1	2	3	4	5	0
6. Seeing the steps of the bus when climbing in or out?	1	2	3	4	5	0
7. Recognizing the face of a person standing near you?	1	2	3	4	5	0
8. Locking or unlocking the door?	1	2	3	4	5	0
9. Doing your usual work either in the house or outside?	1	2	3	4	5	0
10. Doing your work up to your usual standard?	1	2	3	4	5	0
11. Searching for things at home?	1	2	3	4	5	0
12. Seeing differences in colours?	1	2	3	4	5	0
13. Making out differences in coins or notes?	1	2	3	4	5	0
14. Going to the toilet?	1	2	3	4	5	0
15. Seeing the level in the container when pouring?	1	2	3	4	5	0

SID: Date:

D12. Because of your eye problems do you…

	No problem at all	Slight problem	Some problem	A lot	Cannot do this because of your vision
1. Feel frightened to go out at night	1	2	3	4	0
2. Enjoy social functions less?	1	2	3	4	0
3. Feel ashamed that you can't see?	1	2	3	4	0
4. Feel you have become a burden on others?	1	2	3	4	0
5. Feel frightened that you may lose your remaining vision?	1	2	3	4	0

D13. *Ask each of the following questions and tick the appropriate box*

	No problem at all	Slight problem	Some problem	A lot
1. Do you have reduced vision?	1	2	3	4
2. Are you dazzled in bright light?	1	2	3	4
3. Is your vision blurred in sunlight?	1	2	3	4
4. Does bright light hurt your eyes?	1	2	3	4
5. Do you close your eyes because of light from vehicles?	1	2	3	4
6. Does light seem like stars?	1	2	3	4
7. Do you have blurred vision?	1	2	3	4

D14. Have you ever had cataract surgery?

Read all responses

- Yes, both eyes 1☐
- Yes, one eye 2☐
- No 3☐ ►
- Don't know 89☐ ►

If no, Skip to D16

D15. Which year did you have the surgery? ☐☐☐☐

If the elder has had more than one cataract surgery, please put the year of the most recent surgery.

Don't know 89☐

D16. Can you hear a person at his or her normal volume?

Read all responses

Easily	1	☐
With some difficulty	2	☐
With a lot of difficulty	3	☐
Unable to do it	4	☐

D17. When was the last time you had your teeth checked by a dentist?

Never	8	☐
Less than one year ago	1	☐
1 – 2 years ago	2	☐
2 – 5 years ago	3	☐
More than 5 years ago	4	☐
Has no teeth	5	☐
Don't know	89	☐

D18. During the last month how often have you had to eat less or change what you eat because of problems with your teeth?

Read all responses

Often	1	☐
Sometimes	2	☐
Never	3	☐

D19. In the last 12 months have you suffered any injuries?

Yes	☐
No	☐ ▶ *Skip to D21*

D20. If yes, what type of injury did you suffer?

Tick all that are true

Burn	1	☐
Scald (hot water burn)	2	☐
Fall causing bruises or broken bones	3	☐
Poisoning	4	☐
Road traffic injury	5	☐
Near drowning	6	☐
Animal bite	7	☐
Struck / hit by person or object	8	☐

SID: Date:

D21. For each of the following activities, please tell me if you can do it yourself without difficulty, if you need help, if you are unable to do it at all, or it does not apply to you because you don't do that activity.
Circle the number for each box, and then add all the numbers for the score

A.

	Self, without difficulty	Needs help	Unable to do it at all	Does not apply
Stand up from sitting on a chair	0	1	2	
Stand up from sitting on the floor	0	1	2	
Bend, stoop or bow	0	1	2	
Score for mobility				

B.

Eating	0	1	2	
Dressing or undressing	0	1	2	
Take care of appearance (combing hair / shaving)	0	1	2	
Walking	0	1	2	
Get in and out of bed	0	1	2	
Body washing	0	1	2	
Using the toilet	0	1	2	
Score for Dependency in Activities of Daily Living				

C.

Use the telephone	0	1	2	
Able to take a bus	0	1	2	
Shop for food	0	1	2	
Prepare meals	0	1	2	
Sweep the floor or yard, clean the house	0	1	2	
Take medication in right dose at right time	0	1	2	
Manage money	0	1	2	
Score for Dependency in Instrumental Activities of Daily Living				

D22. How concerned are you about falling when:

	Not at all concerned			Very concerned
A. Getting dressed or undressed?	1	2	3	4
B. Taking a bath or shower?	1	2	3	4
C. Getting in or out of a chair?	1	2	3	4
D. Going up or down stairs?	1	2	3	4
E. Reaching for something above your head or on the ground?	1	2	3	4
F. Walking up or down a slope?	1	2	3	4
G. Going out to a social event (e.g. religious service, family gathering)?	1	2	3	4

SID: Date:

Section E. Quality of life

E1. Please listen to each statement carefully and decide to what extent you agree that it is characteristic of your feelings and behaviour.

	Strongly disagree	Disagree	No opinion	Agree	Strongly agree
1. I like to be with people	1	2	3	4	5
2. I prefer working with others rather than alone	1	2	3	4	5
3. I welcome the opportunity to mix socially with people	1	2	3	4	5
4. I find people more stimulating than anything else	1	2	3	4	5
5. I'd be unhappy if I were prevented from making many social contacts	1	2	3	4	5

E2. 1. Are you basically satisfied with your life? Yes 1 ☐ No 2 ☐

2. Do you feel that your life is empty? Yes 1 ☐ No 2 ☐

3. Do you often get bored? Yes 1 ☐ No 2 ☐

4. Are you in good spirits most of the time? Yes 1 ☐ No 2 ☐

5. Do you feel happy most of the time? Yes 1 ☐ No 2 ☐

6. Do you often feel helpless? Yes 1 ☐ No 2 ☐

7. Do you think that most people are better off than you? Yes 1 ☐ No 2 ☐

E3. *Please read each question to the participant. Ask them to assess their feelings, and circle the number on the scale that gives the best answer for them for each question. Where necessary, the questions should be answered in relation to the past two weeks.*

	Not at all	A little	Somewhat	Very much	Extremely
1. How satisfied are you with your health?	1	2	3	4	5

The following questions ask about **how much** you have experienced certain things in the last two weeks.

	Not at all	A little	Somewhat	Very much	Completely
2. How satisfied are you with your level of vision and hearing?	1	2	3	4	5
3. To what extent do you feel that physical pain and discomforts interfere with your activities?	1	2	3	4	5
4. To what extent do you need any medical treatment to function in your daily life?	1	2	3	4	5

	Not at all	Slightly	A moderate amount	Very much	Extremely
5. How much do you enjoy life?	1	2	3	4	5
6. To what extent are you worried about your future?	1	2	3	4	5
7. To what extent are you affected by being dependent on others for your requirements?	1	2	3	4	5

	Not at all	A little	Somewhat	Very much	Always
8. Have you been lonely during the past two weeks?	1	2	3	4	5

SID: Date:

	Not at all	Slightly	A moderate amount	Very much	Extremely
9. Are you satisfied with the amount of money you have to meet your needs?	1	2	3	4	5

	Not at all	A little	Somewhat	Very much	Always
10. How much time have you spent with 'peace of mind'?	1	2	3	4	5

	Not at all	A little	Somewhat	Very much	Extremely
11. How contented are you with your life (life you have lived, achievements you have had in life and capabilities you have acquired)?	1	2	3	4	5
12. How much self-esteem do you have?	1	2	3	4	5
13. How useful have you been to your family?	1	2	3	4	5
14. To what extent are you happy with the responsibilities placed on you by family and society?	1	2	3	4	5

The following questions ask you to say how satisfied you have felt about various aspects of your life over the past two weeks.

	Not at all	A little	Somewhat	Very much	Extremely
15. How satisfied were you with the preference, quality and quantity of food received?	1	2	3	4	5
16. How satisfied were you with the opportunities you had for fun and enjoyment?	1	2	3	4	5
17. How satisfied were you with the opportunities for rest?	1	2	3	4	5

	Not at all	A little	Somewhat	Very much	Extremely
18. How satisfied were you with the amount and quality of sleep you had?	1	2	3	4	5
19. How satisfied were you with the time spent with your children, grandchildren and friends?	1	2	3	4	5
20. How satisfied are you with the recognition you receive from family and society?	1	2	3	4	5
21. How satisfied are you with your ability to perform your day to day work?	1	2	3	4	5
22. How satisfied are you with your ability to perform essential activities of daily functioning such as eating, using the toilet, grooming, washing and dressing?	1	2	3	4	5
23. How satisfied are you with the opportunities, access and the quality of health care received?	1	2	3	4	5
24. How satisfied are you with the comforts at your place of living?	1	2	3	4	5
25. How satisfied are you with the opportunities and time you get to engage in religious and meritorious activities?	1	2	3	4	5
26. How satisfied are you with your personal relationships?	1	2	3	4	5
27. How satisfied are you with you ability and opportunity to take decisions and action regarding yourself?	1	2	3	4	5

SID: Date:

The following question refers to how often you have felt or experienced certain things during the last two weeks.

	Never	Seldom	Quite often	Very often	Always
28. How often have you had negative feelings such as feeling blue, despair, anxiety and depression?	1	2	3	4	5
	Not at all	A little	Somewhat	Very much	Extremely
29. To what extent have you been affected in your activities, by the death or separation of your spouse or a family member (which occurred during the past year)?	1	2	3	4	5
	Very poor	Poor	Neither poor nor good	Good	Very good
30. Considering all of the above how would you rate your 'quality of life'?	1	2	3	4	5

E4. In general, how happy do you feel, on a scale of 0 – 10 where 0 is least happy and 10 most happy? *Circle the number on the scale*

0 1 2 3 4 5 6 7 8 9 10

Section F: Physical measurements

Before I ask you more questions, I would like to measure your blood pressure, and pulse, your weight, height, waist, hips, upper arm and arm span, and check your eyesight.

F1. First I would like to measure your blood pressure and pulse rate. Please stay seated, and once I put this on your wrist keep it steady and at the level of your heart. We will need to take the blood pressure reading and pulse three times. It will squeeze your wrist a bit, but won't hurt. Relax.

Refused	97☐
Not able	98☐

**Time 1** Systolic (mmHg)	☐☐☐
Diastolic (mmHg)	☐☐☐
Pulse rate / minute.	☐☐☐

Ask the respondent to release the arm and relax. Wait for one minute before time 2. Do not ask the respondent questions.

Now we can get your second measurement for your blood pressure.

**Time 2** Systolic (mmHg)	☐☐☐
Diastolic (mmHg)	☐☐☐
Pulse rate / minute	☐☐☐

Again, remind the respondent to relax. Meanwhile, when waiting to take the third measurement, you can locate and measure out a 4 metre length to prepare for the vision test.

Now we can get your third measurement for your blood pressure.

**Time 3** Systolic (mmHg)	☐☐☐
Diastolic (mmHg)	☐☐☐
Pulse rate / minute	☐☐☐

F2. I would now like to measure how tall you are. Please put your feet and heels close together, stand straight and look forward standing with your back, head and heels touching the wall. Look straight ahead.

Measured height (cms)	☐☐☐.☐
Refused	97☐
Not able	98☐

F3. Now we want to measure your weight. Please step on this scale.

Measured weight (Kgs)	☐☐☐.☐
Refused	97☐
Not able	98☐

F4. Now we want to measure around your waist.

Identify the top of the hip bone - and make sure the tape measure is parallel to the floor all the way around the body.

Waist circumference (cms)	☐☐☐.☐
Refused	97☐
Not able	98☐

F5. Next we will measure around your hips.

Measure at the maximum circumference of the hips - and make sure the tape measure is parallel to the floor all the way around the body

Hip circumference (cms)	☐☐☐.☐
Refused	97☐
Not able	98☐

F6. Now I will measure around your left upper arm.

Use your MUAC tape to measure around the left upper arm at the mid-point between the tip of the shoulder and the tip of the elbow.

MUAC (cms)	☐☐.☐
Refused	97☐
Not able	98☐

F7. Next we will measure your arm span.

Please stand against the wall with your arm extended sideways at a 90 degree angle.
Measure from one tip of the middle finger to the U-point.

Half arm span (cms)	☐☐☐.☐
Refused	97☐
Not able	98☐

Now we are going to check your vision. First I am going to test your distance vision.

If the elder is wearing spectacles, ask them to keep them on for this test. Follow the instructions in the manual carefully. Check that the light level is satisfactory. Look at the chart from a distance of 4 metres and check that you can read the 0.0 line yourself.

F8. First I am going to test your distance vision with your right eye. Would you please cover your left eye with the occluder. Please start at the top line of the chart and tell me which way the Es are pointing. Read each line in turn.

Distance Vision – Right Eye ☐.☐☐

F9. Now cover your right eye so I can test your left eye. Please start at the top line of the chart and tell me which way the Es are pointing. Read each line in turn.

Distance Vision - Left Eye ☐.☐☐

If the elder cannot read the line 0.5, check their vision through the pinhole. If they can read line 0.5 or the lines below it then measure their near vision next ***(F12.).***

Now I would like to see if your vision would be better with spectacles.

F10. First I am going to test your right eye. Would you please cover your left eye. Please read the first line of the chart. Now look through the pinhole. Please start at the top line of the chart and tell me which way the Es are pointing. Read each line in turn.

Distance Vision with pinhole - Right Eye ☐.☐☐

F11. Now I am going to test your left eye. Would you please cover your right eye. Please read the first line of the chart. Now look through the pinhole. Please start at the top line of the chart and tell me which way the Es are pointing. Read each line in turn.

Distance Vision with pinhole - Left Eye ☐.☐☐

- *If the distance visual acuity score is equal to or more than 0.5 in either eye, and there is no improvement looking through the pinhole - refer for assessment by ophthalmologist (yellow form).*

- *If the visual acuity score improves to 0.5 or less when looking through the pinhole in either eye it is likely that vision can be improved with spectacles – refer for assessment by an optometrist (blue form).*

SID:	Date:

Next, check the near vision. First check the light level again by checking that you can read the near vision chart line 0.0 from 40 cms (using your 40cms measuring stick). With the elder sitting comfortably, place the rubber end of the measuring stick against the outer corner of their right eye and the other end on the near vision chart.

F12. Now I would like to test your near vision. Looking with both eyes, please start at the top line of the chart and tell me which way the Es are pointing. Read each line in turn.

Near Vision ☐.☐☐

Next you will check for cataract. Take the elder to a dimly lit room. Explain that you would like to look in each eye in turn with the light.

Look at the pupil of each eye in turn with the ophthalmoscope / torch from a distance of about 30 cms. The pupil should look red. If the lens has a cataract the light will not pass through to the retina at the back of the eye, so you will not see the red reflex.

F13. Right eye – red reflex
Yes 1☐
No 2☐

F14. Left eye – red reflex
Yes 1☐
No 2☐

If there is no red reflex in one or both eyes record 'possible cataract' on a yellow referral form and give it to the elder. Tell the elder that they may have a cataract and you will refer them for a further assessment from an eye care professional.

Section G. Family and community participation

The next questions are about your activities, your family and your community.

G1. In your community are there any organized social activities that are for older people? For example, activities might be organized by a Community Based Organisation (CBO), Women's Group or religious leader?

Yes	1☐	
No	2☐	▶ Skip to G3
Don't know	89☐	

G2. What type of activity is it? ……………………………………………………

G3. Is there an Elders' Committee in your Grama Niladari Division?

Yes	1☐	
No	2☐	▶ Skip to G6
Don't know	89☐	

G4. Have you attended any meetings of the Elders' Committee?

Yes	1☐	
No	2☐	▶ Skip to G6

G5. How many meetings of the Elders' Committee have you attended in the past 3 months? ☐☐

G6. In the last year, have you taken part in any social activities (for any age group) in your community? *Read all responses and tick all that apply.*

No	1☐	▶ *Skip to G8*
Community Based Organisation (CBO)	2☐	
Women's Group	3☐	
Youth or Children's Group	4☐	
Temple / Church activities	5☐	
Informal activities with friends	6☐	
Activities with elders	7☐	
Sporting activity	8☐	
Other ………………………………………	9☐	

SID: Date:

G7. In the last year, how often did you take part in the activities you mentioned?
Ask only about the activities mentioned in G6. Tick whichever is closest.

	Daily	Once a week	Once a month	Every six months	Once a year or less
Community Based Organisation					
Women's Group					
Youth or children's Group					
Temple / church activities					
Informal activities with friends					
Activities with elders					
Sporting activity					
Other					

G8. Do you currently do any paid or income-generating work?

Yes ☐

No ☐ ▶ Skip to G10

G9. For how many hours do you do paid or income -generating work each week, on average? ☐☐

If the elder works in an irregular way, help them to work out an average for a week

G10. For how many hours do you work at unpaid work, such as cooking, cleaning, gardening, or child care each day, on average? ☐☐

G11. Do you have a friend or friends that you feel comfortable with, that you can talk to about private matters, or can call on for help?

Yes 1☐

No 2☐

G12. How long does it take to travel to Nuwara Eliya / Walapane?

Less than one hour 1☐

1 – 2 hours 2☐

3 – 4 hours 3☐

5 – 6 hours 4☐

Don't know 89☐

G13. In the last 2 months how often have you visited the town?

Not at all	1	☐
Once or twice	2	☐
Often	3	☐

G14. If you became dependent, would you have someone to look after you?

Yes	1	☐
No	2	☐

G15. In general, do you feel safe and secure in your home?

Yes	1	☐
No	2	☐

G16. In general, do you feel safe and secure in your community?

Yes	1	☐
No	2	☐

G17. Please indicate for each of the statements, the extent to which they apply to your situation, the way you feel now. *Circle the response and write that number (0 or 1) in the score column.*

				Score
A. I experience a general sense of emptiness.	No (0)	More or less (1)	Yes(1)	
B. I miss having people around.	No (0)	More or less (1)	Yes(1)	
C. Often, I feel rejected.	No (0)	More or less (1)	Yes(1)	
D. There are plenty of people that I can lean on in case of trouble	No (1)	More or less (1)	Yes(0)	
E. There are many people that I can count on completely	No (1)	More or less (1)	Yes(0)	
F. There are enough people that I feel close to	No (1)	More or less (1)	Yes(0)	

SID: Date:

Section H. Health-care seeking behaviour

The next questions are about health care.

H1. If you were feeling ill, for example, with fever, cough with phlegm, and chest pain, who would you go to for advice and treatment?

Do not read out the responses - tick all that the elder mentions

Traditional healer	1	☐
Another elder	2	☐
Religious leader	3	☐
EMA	4	☐
PHM	5	☐
Hospital clinic	6	☐
Private clinic	7	☐
No one	8	☐
Don't know	89	☐
Other	9	☐

………………………………………………………………………..

H2. What difficulties do you face in attending a dispensary, clinic or hospital?

Do not read out the responses - tick all that the elder mentions

Transport	1	☐
No one to go with me	2	☐
Can't afford to pay	3	☐
Worried about long waiting times	4	☐
Worried about health staff attitudes	5	☐
Prefer to take home remedies	6	☐
Prefer to see a traditional healer	7	☐
Other	9	☐
………………………………….…………		
Don't know	89	☐

H3. How often in the past month have you visited a hospital, clinic or dispensary? ☐☐

H4. How often in the past month have you visited an ayurvedic doctor or clinic? ☐☐

H5. How much do you estimate that you have spent on health care in the past month? (e.g. to pay for medicines, supplies, investigations, fees)?

Don't know	89	☐
Nothing	1	☐
Rps 1 – 100	2	☐
Rps 101 – 500	3	☐
Rps 501 – 1000	4	☐
Rps 1001 – 2000	5	☐
Rps 2001 – 3000	6	☐
Rps 3001 – 4000	7	☐
Rps 4001 – 5000	8	☐
Other	9	☐

……………………………………

H6. In the last year have any health or social welfare staff visited you at home?

Yes ☐

No ☐ ▶ Skip to I1

H7. Which of the following have visited you in the past year? *(Read all responses and tick all that apply)*

Public Health Inspector	1	☐
Public Health Midwife or Estate Midwife	2	☐
Estate Medical Assistant	3	☐
Social Welfare Officer	4	☐
Elders' Rights Protection Officer	5	☐

SID: Date:

Section I. Health risk factors

The next questions are about your food.

I1. How often do you eat meat, egg or fish?

Read all responses

Every day	1	☐
At least once a week	2	☐
Occasionally	3	☐
Never	4	☐

I2. How many servings of vegetables do you eat on a typical day? ☐☐

(Show picture card) Don't know 89☐

I3. How many servings of fruit do you eat on a typical day? ☐☐

(Show picture card) Don't know 89☐

I4. Have you tried to reduce the amount of salt in your diet?

Yes	1	☐
No	2	☐

I5. In the last 12 months, were you ever hungry but didn't eat because there was not enough money to buy food?

Read all responses

Almost every month	1	☐
Some months, but not every month	2	☐
Only in 1 or 2 months	3	☐
Never	4	☐

Now I am going to ask you about physical activity.

I6. Thinking of the last month, how much time have you spent sitting down on a typical day?

Hours and minutes ☐☐:☐☐

I7. Thinking of the last month, in a typical week on how many days do you do vigorous activity (that raises your heart beat and your breathing a lot) as part of your work or your leisure time? Days ☐

I8. Thinking of the last month, how much time did you spend doing vigorous activity on a typical day? Hours and minutes ☐☐:☐☐

I9. Thinking of the last month, in a typical week on how many days did you do moderate intensity activity (that raises your heart beat and your breathing a little) as part of your work or your leisure time? Days ☐

I10. Thinking of the last month, how much time do you spend doing moderate activity on a typical day? Hours and minutes ☐☐:☐☐

I11. What is your usual source of drinking water?

Tap in the home	1☐
Tap outside – closer than 50 yards	2☐
Tap outside – further than 50 yards	3☐
River	4☐
Well	5☐
Other	9☐
………………………………………	

I12. How long do you spend collecting water each day?

0 – someone else fetches the water	1☐
0 – tap in the home	2☐
5 – 30 minutes	3☐
half to one hour	4☐
one – two hours	5☐
more than two hours	6☐

I13. What type of toilet do you use?

Modern water-flush toilet	1☐
Pour-flush pit latrine	2☐
Pit latrine	3☐
No toilet	4☐
Other	9☐
………………..………………	

SID: Date:

**I14.**	How many people use the toilet you use?	Just your family or household	1☐
	Read all responses	Your household and one other	2☐
		Several households	3☐
**I15.**	Are you able to bathe as often as you would like?	Yes	1☐
		No	2☐
**I16**.	Can you get to a tap easily?	Yes	1☐
		No	2☐
**I17**.	Do you have hot water for washing?	Yes	1☐
		No	2☐
**I18**.	What type of stove does your household use for cooking or boiling? *(Show picture card)*		
		Open fire/ Three stones	1☐
		Surrounded fire	2☐
		Improved single pot stove	3☐
		Anagi-style	4☐
		Griddle stove/ Kerosene	5☐
		Gas stove	6☐
		Electric stove	7☐
		Other	9☐
		....	
**119**.	Do you do the cooking….?	Every day	1☐
	Read out the responses	Most days	2☐
		Sometimes	3☐
		Never	4☐
**I20.**	Is there a chimney in the kitchen?	Yes	1☐
		No	2☐
**I21.**	Did anyone smoke cigarettes or beedis in the house during the last two days?	Yes	1☐
		No	2☐

I22. Do you chew betel?

Yes	1☐	
No	2☐	▶ *Skip to I24*

I23. Do you add tobacco to the betel mix?

Yes	1☐	
No	2☐	

I24. Do you smoke cigarettes or beedis?

Yes	1☐	
No	2☐	▶ Skip to I28

I25. How many do you smoke on average in one day? ☐☐

I26. In the past year have you thought about stopping smoking?

Yes	1☐	
No	2☐	

I27. In the past year have you tried to stop smoking?

Yes	1☐	
No	2☐	▶ Skip to I30

I28. Did you smoke cigarettes or beedis daily in the past?

Yes	1☐	
No	2☐	▶ Skip to I30

I29. How long ago did you stop smoking daily?

Years and months	☐☐.☐☐
Don't know	89☐

I30. In the last 12 months, how often did you drink alcohol?

Never	1☐
Less than once a month	2☐
1-3 days per month	3☐
1 or more days per week	4☐

SID: Date:

I31. Have you ever tried to cut down on drinking alcohol?

Yes 1☐

No 2☐

Section J. Access to telephone, TV, radio, newspapers and magazines

J1. Is there a TV that you can watch if you want to?
- Yes 1☐
- No 2☐

J2. Are you able to listen to a radio?
- Yes 1☐
- No 2☐

J3. Are you able to read a newspaper?
- Yes 1☐
- No 2☐

J4. Do you have your own mobile phone?
- Yes 1☐
- No 2☐

J5. Do you have access to someone else's mobile phone?
- Yes 1☐
- No 2☐

J6. Have you ever used the internet?
- Yes 1☐
- No 2☐ ► Skip to K1

J7. Where did you use it?
Read all responses
- Internet café 1☐
- Friends / relatives 2☐
- At home 3☐
- Other 9☐

……………………………………………………..

J8. How often have you used the internet?
Read all responses
- Only once 1☐
- Only a few times 2☐
- Once a month or more 3☐

SID: Date:

Section K. Economic issues

The next questions are about your economic situation. We know this is a sensitive subject. Please remember that the questionnaire will not have your name on it. I am going to read out categories, so you don't need to tell me an exact amount.

K1. On average, how much money do you get for yourself each month? *Read all responses*

No money at all	1	☐
Below 1000 Rps	2	☐
1001-5000 Rps	3	☐
5001-10,000 Rps	4	☐
More than 10,000 Rps	5	☐

K2. What are your sources of income? *Read all responses, tick whichever are correct*

Paid work	1	☐
Own income generating activity	2	☐
Employees' Provident Fund (EPF)	3	☐
Government pension	4	☐
Government benefit	5	☐
Other family members	6	☐
Other	9	☐

……………………………………

K3. How many people in your household other than yourself received income in the last year?
Do not ask if living alone. ☐☐

K4. What is your estimate of the total income of all household members, from all sources, in the last month? I will read the categories.
Read all responses

0 – 1000 Rps	1	☐
1001 – 5000 Rps	2	☐
5001 – 10,000 Rps	3	☐
10,001 – 15,000 Rps	4	☐
15,001 – 20,000 Rps	5	☐
More than 20,000 Rps	6	☐

K5. Do you have any debt?

Yes	1	☐
No	2	☐ ▶ Skip to K7

K6. How big a worry is the debt to you?

Read all responses

Not a worry at all	1☐
A small worry	2☐
A big worry	3☐

K7. During your working life did you save money for your retirement and old age?

Yes	1☐
No	2☐

K8. Did you receive a lump sum when you retired?

Yes	1☐
No	2☐ ▶ Skip to K11

K9. What has happened to this lump sum? *Read all responses*

Saved in the bank	1☐
All spent	2☐
Most spent	3☐
Some spent	4☐

K10. If all spent – how long after you got it was it spent? (months. weeks) ☐☐.☐☐

K11. In your opinion which would be better? *Read all responses*

To get a lump sum when you retire	1☐
To get a regular fortnightly or monthly pension payment	2☐
Don't know	89☐

Thank you very much for your kind cooperation.

To the interviewer: Now, please complete the interviewer assessment form below, and the Contact Record and Locator Form.

SID: Date:

Interviewer assessment

1. What is your assessment of the elder's cooperation?
   (or family member if interviewed)

Very good	1	☐
Good	2	☐
Moderate	3	☐
Bad	4	☐
Very bad	5	☐

4. What is your evaluation of the accuracy and completeness of the elder's answers?
   (or family member if interviewed)

Very high	1	☐
High	2	☐
Average	3	☐
Low	4	☐
Very low	5	☐

5. Were there any questions with doubtful answers? If so, which were they?

6. Are there any problems or queries that need follow-up or clarification from the Research Coordinator?
